# Uterine Body Stuffing Confirmed by Computed Tomography

**DOI:** 10.5811/cpcem.2017.5.33495

**Published:** 2017-10-18

**Authors:** Michael G. Abesamis, Najeeb Taki, Richard Kaplan

**Affiliations:** * University of Pittsburgh Medical Center, Department of Emergency Medicine, Division of Medical Toxicology, Pittsburgh, Pennsylvania; † Allegheny General Hospital, Department of Emergency Medicine, Pittsburgh, Pennsylvania

## Abstract

A 31-year-old woman presented to an emergency department for suspected vaginal “stuffing” of cocaine. Her physical and pelvic exams were unremarkable despite agitation, tachycardia and hypertension. Abdominal radiograph was concerning for foreign body; transabdominal ultrasound was non-diagnostic. A noncontrast abdominal/pelvic computed tomography (CT) revealed a radiopaque mass within the cervix extending into the uterus. Gynecology was consulted, but the patient refused removal and left against medical advice. Radiographs have varied sensitivity for detecting stuffed foreign bodies; CT is more sensitive and specific. This case suggests that CT is suitable to evaluate for this rare event.

## INTRODUCTION

Body packing allows people to conceal and transport illicit drugs across borders without detection. With increased scrutiny at borders throughout the United States, law enforcement officials have noted that the rates of body packing and stuffing have increased.^1^ More often, body packers will ingest large amounts of drugs through the gastrointestinal tract for later extraction and sale. These include sites such as the mouth, intestine and rectum.^2^

Body stuffing is a unique form of body packing. “Stuffers” hastily place drugs in the mouth, vagina or rectum to quickly conceal available substances from law enforcement. Because of this, the amount of drug in “stuffers” is considerably less than “packers.”^3^ Moreover, compared to “packers,” “stuffers’” drugs are often placed in poorly wrapped packaging that may rupture. Rupture of drug contents into the patient’s system can lead to systemic effects and even death.^3^,^4^,^5^ Because of the packaging differences, if they become symptomatic, effects generally present earlier in body stuffers than with body packers.^6^ Common drugs used for body stuffing include cocaine, heroin, cannabinoids, and methamphetamines.^1^ We present a unique case of a 31-year-old female who was brought to the emergency department (ED) by police with suspected stuffing where the packet was eventually located in the uterus.

## CASE REPORT

A 31-year-old female was brought into the ED by local police. A warrant for a cavity search accompanied the officers, as the patient had been suspected of stuffing cocaine into her vagina when they apprehended her. The patient refused to provide any further details except to deny placing any objects into her vagina or rectum.

Her past medical history was positive for hypertension and noncompliance with her medications. Past surgical history and family medical history was noncontributory. The patient denied alcohol, smoking or drug use, and she had no known drug allergies. On review of systems, the patient denied abdominal pain, chest pain, shortness of breath, pelvic pain, vaginal bleeding or vaginal discharge. The rest of the 10-point review of systems was otherwise negative.

On physical exam, vital signs included a blood pressure of 148/110 mmHg, pulse of 125 beats per minute, respiratory rate of 16 per minute, and pulse oximetry of 100% on room air. She appeared to be a well-developed, well-nourished female in no acute distress. Head was atraumatic, normocephalic, and pupils were equal, round and reactive to light and accommodation. Cardiopulmonary exam was only significant for tachycardia. Her abdominal exam was unremarkable with normal bowel sounds, and no organomegaly, rebound or guarding. Rectal exam revealed no evidence of foreign body. Pelvic exam disclosed normal external genitalia, no vaginal discharge, no cervical motion tenderness and no foreign body seen on speculum exam. The cervical os appeared closed and normal but was not digitally explored. Neurological and psychiatric exam were within normal limits.

**Image 1:**
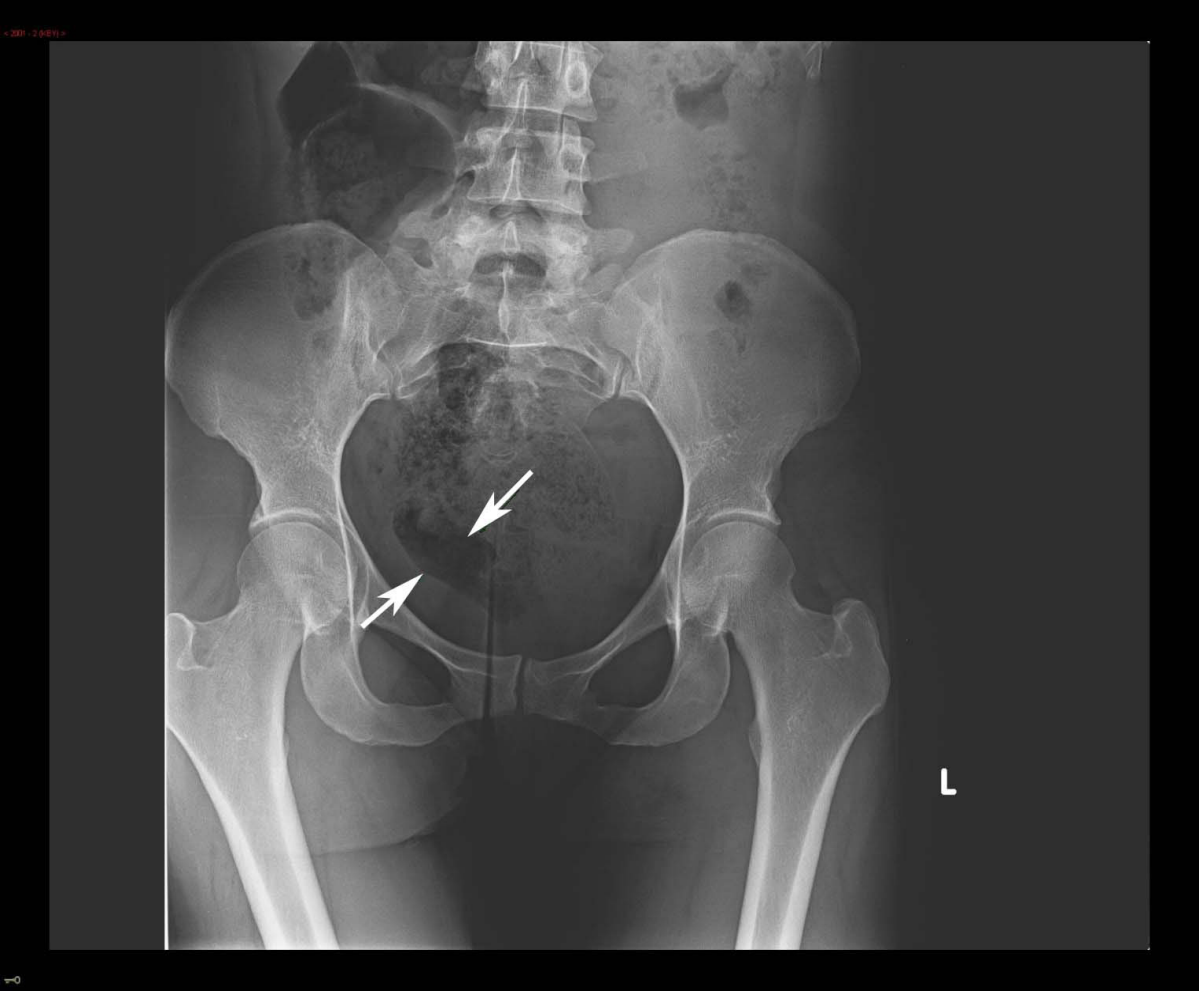
Kidney, ureter, bladder radiograph with arrows showing an area concerning for a possible foreign body in the right hemipelvis

**Image 2:**
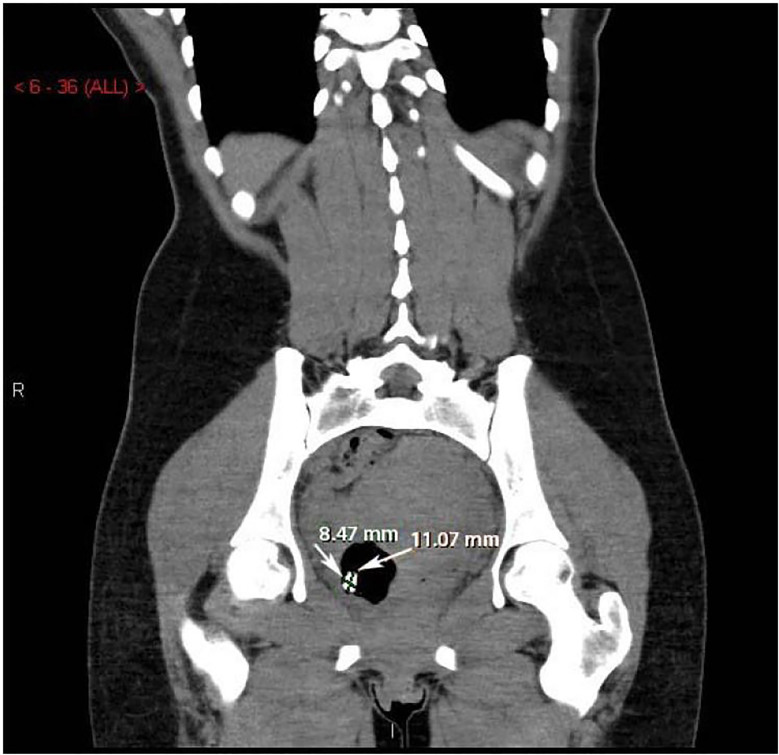
Coronal computed tomography of the abdomen/pelvis with arrows revealing a tablet-like, radiopaque mass within the cervix extending into the uterus.

**Image 3:**
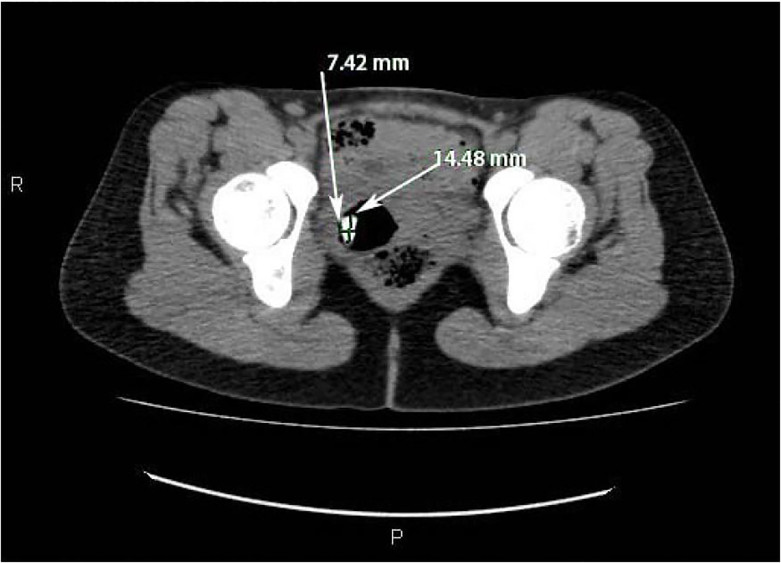
Axial computed tomography of the abdomen/pelvis with arrows revealing a tablet-like, radiopaque mass within the cervix that extends into the uterus.

Urine pregnancy was negative and a kidney, ureter, bladder (KUB) radiograph was initially negative, but over-read by radiology as concerning for foreign body in the right hemipelvis (Image 1). A transabdominal ultrasound was obtained, but was negative. The patient refused a transvaginal ultrasound. After consultation with radiology, a non-contrast computed tomography (CT) of her abdomen/pelvis was performed to further delineate the location of the foreign body, revealing a tablet-like, radiopaque mass within the cervix that extended into the uterus (Images 2 and 3).

Gynecology was consulted for removal of the uterine foreign body. However, the patient refused any further testing or procedures. The hospital attorney was consulted and the emergency physician was informed that the patient could not have the foreign body removed against her volition. The patient signed out of the ED against medical advice. Since no physical evidence could be produced, the patient was released by the police.

## DISCUSSION

This case demonstrates a unique event in relation to illicit substance stuffing. This patient had a suspected cocaine bag in her uterus that we believed she stuffed. To our knowledge, this is the first documented case in the literature. This “stuffing” was concerning as intravaginal toxicity due to stuffing has been described in the past,^4^ and given the vascularity of the uterine bed, toxicity from drug exposure from this site is a significant possibility.

Evaluating which method would be best to assess a similar case was explored. In reviewing the past stuffing/packing literature, we found that radiographs have been reported to have a sensitivity that varies dramatically (47% – 95%) in finding stuffed/packed foreign bodies.^7^ This was thought to be secondary to the different radio-opacities of the substances (cannabis is radiopaque, cocaine is isodense, and heroin in radiolucent) and the packaging materials used. CT is considered to be more sensitive than radiographs in locating foreign bodies.^8^ One study suggests ultrasound as a possible screening tool with a positive predictive value of 97% and an accuracy of 94% in searching for intestinal foreign-body packing,^9^ but it has not been studied in body stuffing, and utility would likely vary on the body cavity involved and the amount of drug placed.

**CPC-EM Capsule**

What do we already know about this clinical entity?

Drug “stuffers” often place poorly wrapped substances in orifices to conceal from law enforcement. Rupture of the packaging can lead to systemic toxicity and death.

What makes this presentation of disease reportable?

We present the first confirmed case of uterine stuffing via computerized tomography.

What is the major learning point?

Uterine stuffing is a rare but possible event which can be missed on physical exam. Given the vascularity of the uterus, systemic toxicity is theoretically possible.

How might this improve emergency medicine practice?

When assessing for uterine stuffing a non-contrast computerized tomography scan of the abdomen and pelvis is adequate to evaluate for this rare event.

In this case, ultrasound was not useful likely due to the uncooperative patient, inability to perform the study transvaginally, and operator-dependent differences. Abdominopelvic CT is considered the most accurate method of diagnosis of body packing/stuffing with sensitivity between 77–100% and specificity of 94–100%.^10^,^11^ Evidence for the use of CT with or without oral contrast in evaluating cases of body packers and stuffers has been limited. However, recent studies suggest that CT without oral contrast is more sensitive and has an equal positive predictive value compared to CT with oral contrast.^12^

“Stuffers” generally wrap drugs in materials such as cellophane, plastic bags, aluminum foil, glassine crack vials, or wax paper,^3^ due to the rapid manner in which they attempt to hide the substances. These are more likely to rupture or leak; therefore, one must keep a high vigilance especially in cases that have negative clinical findings. Rupture of the contents can lead to disastrous consequences, ranging from drug toxicity to death.^3^,^4^,^5^ When assessing body packers/stuffers, physical exam should include a head-to-toe examination with focus on vital signs, neurological status, pupil size and abdominal exam.^1^ Examination of the rectum and vagina should be attempted as packets may be visible.^3^,^4^ Many clinicians have opted for conservative management in asymptomatic body packers/stuffers.^1^,^6^,^7^ Initially, body packers were taken to the operating room for laparoscopic removal of their drug pellets. Now, a watch- and-wait approach has been advocated to allow spontaneous passage of the drugs, possibly in an intensive care unit setting.^1^,^6^,^7^ Symptomatic body packers may require urgent operative management.^1^,^7^ However, since the amount of drugs in “stuffing” is less, a wait-and-watch approach can also be taken. These patients can be treated symptomatically unless the toxicity from the offending agent is severe.^6^,^7^

## CONCLUSION

The care of a body stuffer or packer can have legal and ethical ramifications. How should one proceed with a patient brought in under suspicion of stuffing an illegal substance? In this case, the patient was accompanied with a warrant for a body cavity search. Beyond a physical exam, non-invasive methods were used during this patient encounter. If there is a concern for possible stuffing, particularly for stuffing into a hard-to-access body cavity such as a uterus, we would recommend a non-contrast abdominopelvic CT as part of the evaluation based on this experience. Administering sedative medications for an invasive procedure with the sole purpose of extracting evidence is not covered under a standard cavity search. With complex and potentially life-threatening situations such as these, we strongly advocate the use of the hospital legal department and ethics committee to help resolve matters.
